# Successful Catheter‐Directed Thrombolysis for a Patient With Intermediate‐High‐Risk Pulmonary Embolism: A Case Report

**DOI:** 10.1002/ccr3.71863

**Published:** 2026-01-30

**Authors:** Mohammadreza Motazedian, Ahoura Salehi Nowbandegani, Zahra Mohammadi, Sina Bazmi

**Affiliations:** ^1^ School of Medicine Fasa University of Medical Sciences Fasa Iran; ^2^ Student Research Committee Fasa University of Medical Sciences Fasa Iran; ^3^ USERN Office Fasa University of Medical Sciences Fasa Iran

**Keywords:** case report, catheter‐directed thrombolysis, computed tomography angiography, interventional cardiology, pulmonary embolism

## Abstract

Acute pulmonary embolism (PE) is a prevalent cardiovascular condition with significant mortality and morbidity. Treatment strategies vary according to risk stratification. While anticoagulation is sufficient for low‐risk patients, high‐risk cases often necessitate systemic thrombolysis (ST) or surgical embolectomy. Catheter‐directed therapy (CDT) has emerged as an alternative for high‐ and intermediate‐high‐risk patients, particularly when ST is contraindicated or poses a high bleeding risk. Through CDT, thrombolytic drugs are locally delivered straight into the pulmonary arteries. Despite promising outcomes in select cases, evidence for CDT remains inconclusive, reflected in its class‐IIa recommendation in current guidelines. We describe a 44‐year‐old male who experienced sudden and worsening dyspnea over 5 days. The patient had a history of smoking, methadone addiction, and a recent motor vehicle accident, with a prior intracranial hemorrhage. Echocardiography revealed right ventricular dilation and systolic dysfunction, and computed tomography pulmonary angiography confirmed massive bilateral pulmonary artery thrombosis, while the patient's troponin level was 996 ng/L (reference < 2), categorizing the patient as intermediate‐high risk. Due to contraindications to systemic thrombolysis and lack of response to anticoagulation, CDT was performed, resulting in rapid improvement in symptoms, oxygenation, and right ventricular function. The patient was discharged without complications. He was transitioned to oral anticoagulation and remained stable at three‐month follow‐up. This case highlights the potential of CDT as an effective and safe treatment for PE patients with intermediate‐to‐high risk who are not candidates for ST, a scenario with limited therapeutic options. CDT's routine use requires validation through additional studies and randomized trials.

## Key Clinical Message

Catheter‐directed therapy (CDT) is a viable treatment for intermediate‐high‐risk pulmonary embolism when systemic thrombolysis is contraindicated. This case highlights CDT's role in rapidly improving symptoms, oxygenation, and right ventricular function without complications, emphasizing its potential as a safer alternative that requires further validation through clinical trials.

## 1 Introduction

Pulmonary embolism (PE) is a prevalent acute cardiovascular disease contributing to significant mortality and morbidity [1]. PE presents variably, from mild symptoms to hemodynamic compromise. Given PE's significant clinical burden, timely and effective treatment strategies are critical. The treatment approach for PE patients varies depending on the risk classification outlined in the guidelines [2].

For low‐risk PE patients, anticoagulants alone are typically used as the primary treatment. In high‐risk cases, systemic thrombolysis (ST) is employed, and if contraindicated or ineffective, embolectomy surgery may be considered [3]. In high‐risk PE patients, the benefits of thrombolysis outweigh the associated bleeding risks. However, in intermediate‐risk patients, the balance between the potential advantages of thrombolysis and the associated bleeding risks is not as clear [4, 5]. The process of making decisions for patients at intermediate risk is more difficult and depends on the patient's specific medical conditions [6].

Systemic fibrinolysis carries a higher risk of hemorrhagic stroke and severe bleeding, with limited survival benefits [7, 8]. Embolectomy surgery, however, is an invasive treatment involving cardiopulmonary bypass and pulmonary artery dissection, which carries its own mortality and comorbidity risks, along with potential complications. In contrast, catheter‐directed therapy (CDT) offers a less invasive interventional approach. With targeted delivery and lower doses of thrombolytic agents, CDT reduces the bleeding risk compared to ST and avoids the complications associated with embolectomy surgery. It can be an option for patients classified as high‐risk or intermediate‐high‐risk when ST is contraindicated or unsuccessful, as recommended by the guidelines [9, 10, 11].

Despite the emergence of CDT approximately two decades ago, there is a scarcity of evidence‐based data regarding its efficacy and safety. Currently, CDT is classified as a class IIa recommendation, and the clear mortality benefit of this approach is yet to be demonstrated. In light of the limited evidence supporting CDT in intermediate‐high‐risk PE and the challenges of managing patients contraindicated for systemic thrombolysis, we present a case demonstrating the successful use of CDT in treating bilateral acute PE.

## 2 Case History/Examination

A 44‐year‐old male patient came to our Emergency Department complaining of burning chest pain and shortness of breath that began suddenly 5 days earlier and progressively worsened over time. The patient had a history of smoking, COPD, methadone addiction, and intracranial hemorrhage due to trauma but no prior history of heart attack, heart failure, or coronary artery disease. Twelve days ago, the patient had been hospitalized for 5 days due to a rib and hip fracture following an accident, during which he was immobile; low‐molecular‐weight heparin (LMWH) therapy had not been administered after discharge, contributing to a higher risk of venous thromboembolism. Upon arrival, the patient exhibited tachycardia with a heart rate of 134 b.p.m. and a blood pressure of 100/60 mmHg. He was afebrile with a temperature of 36.5°C. The patient's blood saturation level of oxygen was 80% while inhaling ambient air but reached 93% with the delivery of oxygen with an O_2_ nasal cannula at a rate of 5 L/min. The patient had a respiratory rate of 24 breaths per minute and an electrocardiogram (ECG) showing sinus tachycardia with ST segment depression in V3‐V6 and a negative T‐wave in V1‐6 and lead III. Additionally, the patient's ECG showed an S1Q3T3 pattern. Laboratory tests revealed an elevated C‐reactive protein level (58 mg/L) (reference range < 6 mg/L), elevated white blood cell count (11.1/nL) (reference range: 4000–11,000/nL), and high‐sensitive troponin T (hs‐TnT) level (996 ng/L) (reference range < 2 ng/L).

## 3 Differential Diagnosis, Investigations, and Treatment

Dyspnea in a young male patient with a history of smoking should be approached systematically. The causes can be categorized as either systemic or organ‐specific. Cardiac causes include vascular, valvular, myocardial, and pericardial. Likewise, lung conditions can be classified into mediastinal, parenchymal, and pleural conditions. Patients with tachycardia, hypoxia, and dyspnea should be evaluated for potential vascular lung issues. In the absence of a history of extended immobility, the diagnosis of pulmonary embolism is frequently missed. Even in patients with normal mobility, a heightened level of suspicion is essential for the accurate and prompt diagnosis of this condition [12].

**FIGURE 1:**
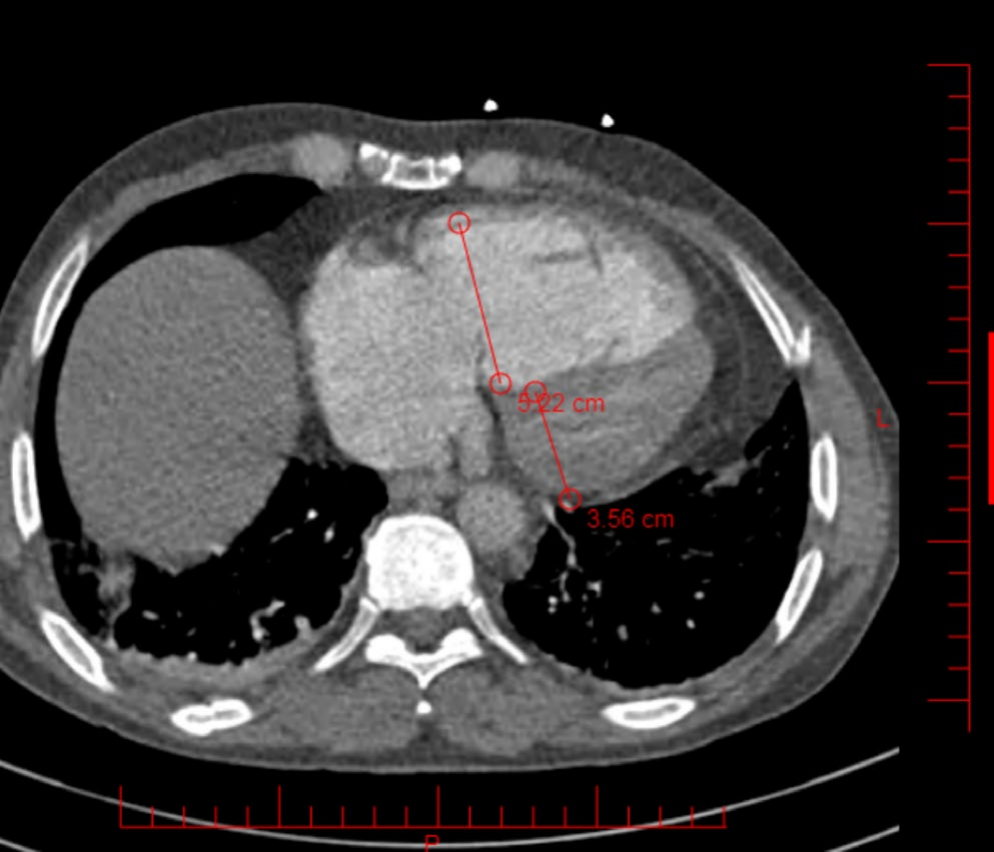
Contrast‐enhanced computed tomography pulmonary angiography (CTPA) demonstrating a massive pulmonary embolism with filling defects in the main pulmonary artery, extending into the left pulmonary artery and its segmental and subsegmental branches, as well as the right pulmonary artery. Measurements of the right ventricular (5.22 cm) and left ventricular (3.56 cm) dimensions suggest right ventricular dilation.

In our case, Echocardiography showed right ventricle dilation with a size of 50 mm, indicative of right ventricular dysfunction and a D‐shaped left ventricle. Left ventricular function was mildly decreased due to abnormal motion of the septum (ejection fraction of 50%). The systolic pulmonary artery pressure (sPAP) was 49 mmHg, with moderate tricuspid regurgitation with a 39 mmHg gradient (TRG). IVC appeared enlarged with normal collapse. Acute PE was the most likely diagnosis. Furthermore, there were no signs of deep vein thrombosis. The Wells score was 6, the PESI score was 144, and the simplified score was 2, placing the patient in a high‐risk category for mortality. Massive bilateral pulmonary emboli were identified through contrast‐enhanced computed tomography pulmonary angiography (CTPA), with simultaneous filling defects observed in both the right and left pulmonary arteries (Figure 1). Consequently, the patient was moved to the cardiac care unit. Due to a previous intracranial hemorrhage, ST was contraindicated; thus, intravenous unfractionated heparin therapy was initiated and titrated to achieve target activated partial thromboplastin time (aPtt).

**FIGURE 2:**
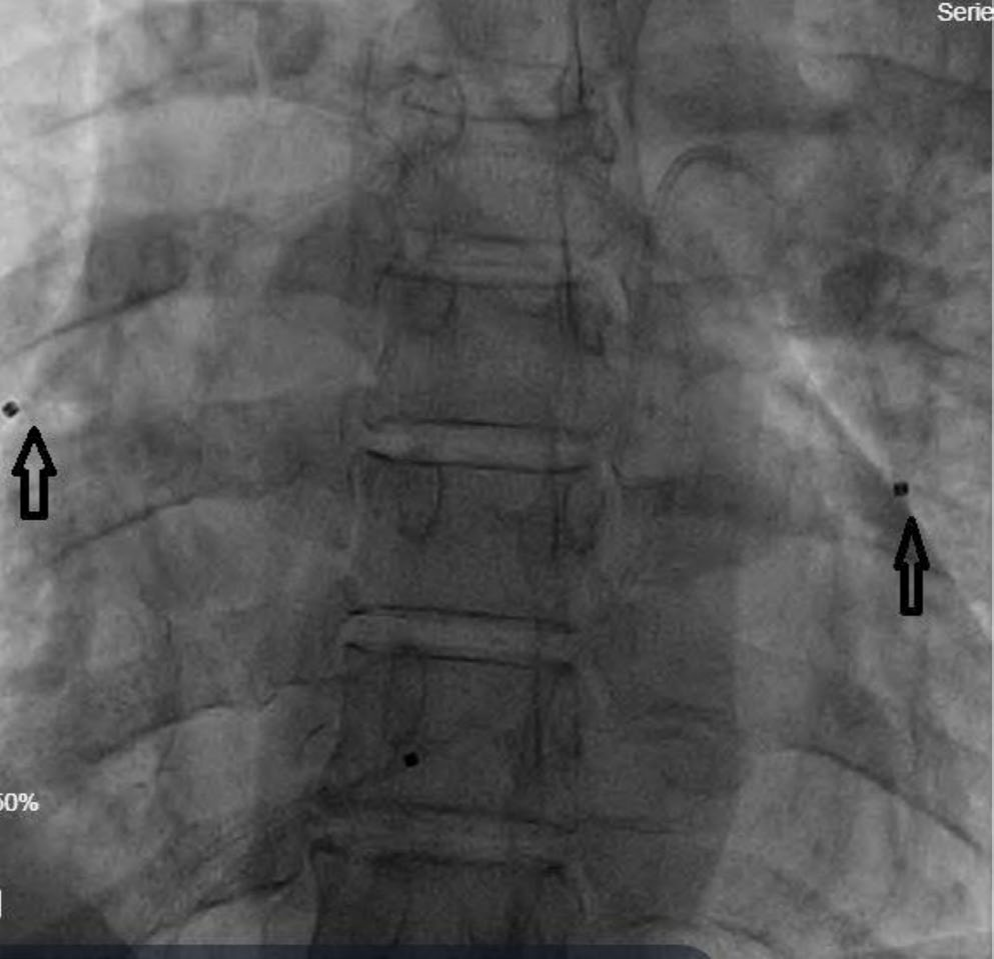
Fluoroscopic image showing the positioning of catheters in the pulmonary arteries for catheter‐directed thrombolysis.

On the third day, follow‐up echocardiography revealed an increase in sPAP to 75 mmHg, the reduced collapse of the IVC, a TRG of 60 mm, and dilation of the right ventricle measuring 55 mm in size. Owing to clinical deterioration in symptoms resulting in worsening shortness of breath, a decrease in blood pressure to 90 mmHg, and the unavailability of a heart surgeon for embolectomy, the cardiology team decided to proceed with CDT. The medical team transferred him to the cardiac catheterization lab. A 6 Fr sheath was inserted to puncture and cannulate the right common femoral vein. A manual injection of iodinated contrast through the femoral venous sheath was used to perform the initial IVC angiography. This step is critical to prevent missing any thrombus in the path of catheter advancements and thus prevent disastrous consequences where the thrombus present in the IVC can be extended to the heart and pulmonary circulation. Two Cragg‐McNamara ev3 Endovascular 5 Fr catheters were inserted using a 0.035 in. × 260 cm exchange length Terumo GLIDEWIRE guidewire (Terumo Corporation, Tokyo, Japan). The catheters were guided to the right atrium, right ventricle, and main pulmonary artery, and subsequently positioned in both pulmonary arteries, as shown in Figure 2. After injecting a 1‐mg bolus of alteplase into the thrombotic regions of the right and left pulmonary arteries, the catheters were left in place for 24 h, with 5 mg in the right catheter and 15 mg in the left catheter. Because a larger thrombus bulk was seen in the left pulmonary artery based on computed tomography pulmonary angiography (CTA), we decided to inject more dosage of alteplase in the left catheter. The patient was observed continuously in the cardiac care unit and intravenous unfractionated heparin (IV UFH) infused contemporaneously. Finally, a total of 20 mg of t‐PA was administered and on post‐operative day (POD) the infusions were stopped and the catheters removed. Following catheter removal, IV UFH therapy was continued.

## 4 Outcome and Follow‐Up

The patient's course during local thrombolysis was uneventful, with gradual increases in blood pressure and oxygen saturation levels and a decrease in heart rate. The following day, the patient's oxygen saturation reached 93 without oxygen supplementation, blood pressure reached 110/80 mmHg, and the heart rate decreased to 85 b.p.m. The patient's echocardiography showed a reduced right ventricular diameter of 42 mm and the sPAP was 48 mmHg. On POD 3, the patient was moved to the general ward and started on oral anticoagulation with rivaroxaban 15 mg twice daily. The patient's clinical course remained uncomplicated, and after 5 days, the patient was discharged with advised smoking cessation. Considering the patient's oxygen saturation level, which improved to 93% with supplemental oxygen, and the patient's history of smoking, a consultation with a pulmonologist was requested. Spirometry was performed, confirming the diagnosis of chronic obstructive pulmonary disease (COPD). The patient received oxygen therapy and was discharged with prescriptions for Ipratropium bromide and Budesonide‐Formoterol inhalers. Rivaroxaban 15 mg was administered twice daily for 3 weeks and then follow‐up echocardiogram demonstrated the right ventricle at 38 mm, and the sPAP was 38 mmHg. At this time, anticoagulation therapy was changed to rivaroxaban 20 mg daily and continued.

After the procedure, the patient was followed up in the outpatient setting. During the subsequent 3 months, the patient's condition remained stable without complications.

## 5 Discussion

PE symptoms differ based on the extent of the thromboembolic obstruction in the pulmonary circulation, the associated vasoconstrictor response, and the right ventricle's capacity to manage the acute pressure overload. To risk‐stratify patients, the American Heart Association categorizes acute PE into three groups: low‐risk, submassive, and massive [13]. The European Society of Cardiology (ESC) uses a similar low‐risk, intermediate‐risk (further divided into high and low), and high‐risk classification system, with the main difference being the ESC's more detailed approach to the intermediate‐risk category [14]. The ESC guidelines incorporate additional clinical factors, such as cardiac biomarkers and the PE Severity Index, alongside right ventricular dysfunction on imaging, to better capture the complexity of the intermediate‐risk group. As per the ESC, signs of hemodynamic instability (e.g., shock, persistent hypotension) along with confirmed PE on imaging are sufficient to classify a patient as high‐risk, while intermediate‐risk PE is indicated through dysfunction of the right ventricle and/or increased cardiac biomarkers without hemodynamic instability [3, 15].

Treatment options for PE include anticoagulation alone, systemic low‐dose thrombolysis, systemic full‐dose thrombolysis, catheter‐directed interventions, and surgical treatment. Anticoagulation is sufficient only for low‐risk patients, while ST is recommended for high‐risk patients. The treatment of intermediate‐risk individuals is more challenging and relies on various factors, such as bleeding risk, institutional expertise, location and extent of thrombosis, and patient characteristics [6, 16]. The lack of evidence supporting the efficacy and safety of existing catheter‐based therapies contributes to the uncertainty surrounding the best treatment for intermediate‐risk PE. The 2019 European Society of Cardiology [3] and the 2021 American College of Chest Physicians guidelines [17] recommend against ST in hemodynamically stable patients with submassive PE and instead suggest anticoagulation. Additionally, the hemodynamic status of patients with PE may evolve over time, necessitating adjustments in therapy [6, 16]. Catheter‐based therapies are an alternative for high‐risk PE patients with contraindications to or inadequate effect of ST (Class IIa, level C) and may be recommended for intermediate‐risk patients at high risk of bleeding (Class IIb, level B) [18]. While ST can rapidly reduce thrombus burden and right ventricular overload, it carries a significant risk of severe bleeding, especially intracranial hemorrhage, which may offset its potential benefits [8]. Surgical embolectomy and CDT are alternative options for intermediate‐ and high‐risk PE patients who experience hemodynamic worsening despite anticoagulation or where thrombolysis is contraindicated or unsuccessful. However, the majority of patients are poor candidates for surgery due to their preoperative condition and comorbidities [10, 11]. Furthermore, the best therapeutic approach remains unclear for patients with intermediate‐ or high‐risk PE on anticoagulation whose hemodynamic condition does not improve or deteriorate [19].

Concerns over the bleeding risks linked to ST have led to increasing interest in alternative management strategies for intermediate‐ and high‐risk PE, including CDTs. Novel catheter‐based approaches, such as catheter‐directed thrombolysis and catheter‐directed embolectomy (CDE), offer effective thrombus removal without ST or surgical treatment risks. Catheter‐directed therapies deliver a catheter into the branches of the pulmonary arteries and thrombus, enabling low‐dose alteplase administration, ultrasound‐assisted thrombolysis, and mechanical thrombus fragmentation and aspiration. The directed thrombolysis catheter was designed to provide a thrombolytic effect similar to ST while reducing bleeding complications by locally delivering a lower dose of the thrombolytic agent [10, 11]. Additionally, CDTs are less invasive than surgical embolectomy and are linked to a reduced incidence of periprocedural morbidity [20]. Preliminary data indicate that CDT achieves a procedural success rate of over 80%. This success is defined by the correction of hypoxemia, hemodynamic stabilization, and survival to hospital discharge. However, its mortality benefits remain unclear. This is largely due to the reliance on observational studies rather than randomized controlled trials [21]. Our case highlights the need for individualized treatment strategies until stronger evidence emerges. The ESC Working Group on pulmonary circulation and right ventricular function and the European Association of Percutaneous Cardiovascular Interventions recently issued a clinical consensus statement proposing algorithms and timelines for CDTs, reflecting the growing clinical and scientific interest in this novel field [22].

A recent meta‐analysis by Planer et al. compared the efficacy and safety of CDT with other treatment options for intermediate‐ or high‐risk PE. The study revealed that CDT was related to a lower risk of death and major bleeding complications than ST, with moderate certainty of evidence. Additionally, when contrasted with anticoagulation alone, CDT was linked to a likely reduction in mortality risk and comparable risk of intracranial hemorrhage. These results suggest that CDT could be considered a frontline therapy for patients with intermediate‐ or high‐risk PE [23]. However, the authors cautioned that much of the existing evidence is based on observational studies, highlighting the need for further exploration of CDT's role, especially in diverse clinical scenarios. Additionally, a meta‐analysis in 2022 suggested that CDT is linked to significantly lower in‐hospital, 30‐, and 90‐day mortality. It also showed a trend toward decreased 1‐year mortality and better RV recovery compared to systemic anticoagulation alone in patients with submassive pulmonary embolism [4]. However, one study found a 10% incidence of major extracranial bleeding requiring transfusion during CDT [24]. Notably, the PEITHO trial compared the use of anticoagulation and ST versus anticoagulation alone. It found that ST significantly reduced death and hemodynamic decompensation but increased the rate of severe intracranial and extracranial bleeding [8]. Given the limitations of the available data, primarily the lack of head‐to‐head randomized controlled trials comparing CDT with full‐dose systemic thrombolytic therapy, the superiority of catheter‐based approaches over systemic thrombolysis remains uncertain.

The routine use of CDT for intermediate‐risk PE patients is not supported by the available literature. In this case, CDT was chosen due to the patient's contraindication to systemic thrombolysis, worsening hemodynamic status, and limited surgical options, demonstrating its potential value in carefully selected intermediate‐risk patients. The case contributes to the growing evidence supporting CDT as a primary treatment for submassive PE. However, randomized controlled trials are necessary to make definitive conclusions about whether CDT is superior to ST. These trials are also needed to determine the most effective and safe method of CDT. Additionally, they will help identify the patient populations that could particularly benefit from catheter‐directed treatment.

## 6 Conclusion

In this study, we report the case of an intermediate‐risk patient who was treated with catheter‐directed thrombolysis, leading to rapid improvement in vital signs and immediate resolution of symptoms, without complications. This case highlights the importance of timely diagnosis, accurate risk stratification, and individualized treatment strategies in improving outcomes for PE patients. The successful use of CDT underscores its potential as a viable option in specific scenarios; though further studies are needed to refine its indications and long‐term benefits.

## Author Contributions

**Mohammadreza Motazedian:** conceptualization, project administration, supervision, validation. **Ahoura Salehi Nowbandegani:** conceptualization, project administration, resources, supervision, validation. **Zahra Mohammadi:** data curation, investigation, resources, writing – original draft, writing – review and editing. **Sina Bazmi:** data curation, investigation, validation, writing – review and editing.

## Funding

The authors have nothing to report.

## Ethics Statement

As a single‐case report with the patient's signed consent, no other ethical review was required.

## Consent

The authors confirm that they have obtained written informed consent from the patient, who has agreed to have his clinical information and images reported in the journal. The patient acknowledges that his name and initials will not be disclosed, and reasonable measures will be taken to protect his privacy. However, complete anonymity cannot be guaranteed.

## Conflicts of Interest

The authors declare no conflicts of interest.

## Data Availability

The data supporting this study's findings are available from the corresponding author upon reasonable request.
